# The Role of Tumor Microenvironment in Triple-Negative Breast Cancer and Its Therapeutic Targeting

**DOI:** 10.3390/cells14171353

**Published:** 2025-08-30

**Authors:** Ana Vuletić, Katarina Mirjačić Martinović, Vladimir Jurišić

**Affiliations:** 1 Department of Experimental Oncology, Institute of Oncology and Radiology of Serbia, Pasterova 14, 11000 Belgrade, Serbia; 2 Faculty of Medical Sciences, University of Kragujevac, P.O. Box 124, 34000 Kragujevac, Serbia

**Keywords:** triple negative breast cancer, tumor microenvironment, cancer associated fibroblasts

## Abstract

Triple-negative breast cancer (TNBC) is an aggressive subtype of breast cancer characterized by rapid proliferation and infiltration of immune cells into tumor microenvironment (TME). The treatment of TNBC still remains challenging due to the lack of expression of effective molecular targets pertaining to the tumor cell itself. In TNBC standard of care, therapies such as chemotherapy, together with recently introduced immunotherapy with checkpoint inhibitors, often do not result in durable clinical response. Therefore, better understanding of complex interactions between tumor cells, immune cells, and stromal cells mediated by multiple cytokines, chemokines, enzymes, and metabolites in TME is crucial for understanding the mechanisms that underlie tumor cell immune evasion strategies. The aim of this review is to give comprehensive overview of immune cell network and their interactions with cells in TME and possibilities for therapeutic targeting of TME in TNBC. We discuss cancer-associated fibroblasts (CAFs) as an important recently characterized player in TNBC with respect to their role in interactions with immune cells and their impact on tumor invasion. Based on the recently accumulated knowledge, therapies targeting immune suppressive mechanisms and CAF-related tumor-promoting mechanisms in TME hold great potential for clinical evaluation in TNBC.

## 1. Introduction

Tumor microenvironment (TME) or tumor stroma is a complex biological network in which malignant cells coexist with a variety of nonmalignant cells, including immune cells, tumor-associated fibroblasts (CAFs), mesenchymal stem cells (MSCs), tumor-associated adipocytes, endothelial cells, and pericytes in glycoprotein-rich extracellular matrix (ECM). The crosstalk between malignant cells and TME contributes to epithelial-to-mesenchymal transition (EMT), acquisition of invasive phenotype, increased motility of tumor cells, and subsequent formation of secondary tumor deposits [1].

Breast cancer is one of the most prevailing malignancies [2]. Triple-negative breast cancer (TNBC) is characterized by the lack of expression of estrogen receptor α (ER-α), progesterone receptor (PR), and the absence of expression/amplification of human epidermal growth factor receptor 2 (HER2). TNBC exhibits a higher proliferation rate and higher incidence of metastases compared to other breast cancer types [3]. The heterogeneous molecular characteristics of TNBC reduce the options for targeted therapies, making this tumor particularly aggressive and resulting in poor prognosis [4]. Accordingly, systemic chemotherapy based on anthracyclines and taxanes remains the primary treatment option [5]. In the past decade, based on studies involving genomic analyses, TNBC was classified into distinct molecular subtypes that were further refined according to the immunomodulatory gene expression profile attributed to the tumor infiltrating lymphocytes (TILs) and stromal cells [6,7]. The classification proposed subtypes displaying distinct clinical and pathological characteristics with disease prognosis differing significantly between the subtypes, and each subtype showing a specific pattern of gene expression [8,9]. However, the classification of TNBC due to the complexity and heterogeneity of the tumor, remained without the true consensus. Nevertheless, the four subtypes identified based on gene expression profiles named basal-like immune activated (BLIA), basal-like immune suppressed (BLIS), luminal androgen receptor (LAR), and mesenchymal (MES) proposed by Burstein et al. are the most thoroughly validated [8,10]. In one study TNBC was classified according to genomic immune profile in TME [11].

Recently, immunotherapy based on the inhibition of immune checkpoint (IC) molecules, most notably of the programmed cell death receptor (PD)-1 and cytotoxic T lymphocyte antigen (CTLA)-4, has shown potential to enhance immune response against tumor cells and thereby modulate TME [12,13]. In this sense, PD-/PD-L1 blockade with Food and Drug Administration (FDA)-approved monoclonal antibodies such as anti-PD-1 (pembrolizumab, nivolimab, cemiplimab, camrelizumab), anti-PD-L1 (atezolizumab, durvalumab, avelumab), and CTLA-4 blockade with ipilimumab, were introduced in clinical trials for breast cancer in TNBC and have shown therapeutic potential [14]. However, monotherapy with IC inhibitors resulted in limited clinical benefit as tumor cells are prone to immune escape and drug resistance due to highly dynamic interplay between the cells in TME. In the context of available therapeutic options including chemotherapy, radiotherapy and immunotherapy, there is a constant need for better understanding of cellular and molecular components of TME and their interactions that could be targeted in order to overcome therapeutic limitations and improve treatment outcome.

## 2. Immune Cells in TME

**Figure 1:**
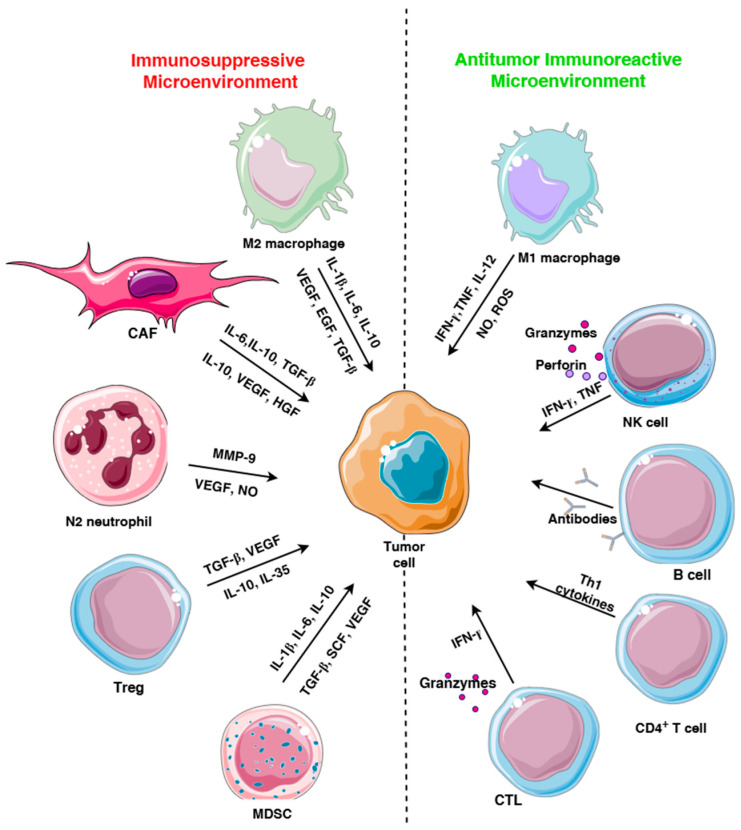
Immunosuppressive and immunoreactive cells in tumor microenvironment (TME). Cells in TME: M2 macrophages, cancer-associated fibroblasts (CAF), N2 subset of neutrophils, regulatory T cells (Treg), myeloid-derived suppressor cells (MDSC), M1 macrophages, natural killer (NK) cells, cytotoxic T lymphocytes (CTL), CD4 helper T cells, and B lymphocytes.

Immune cells in TME include immunoreactive cells such as CD8 cytotoxic T lymphocytes (CTL)s, CD4 helper T cell, B lymphocytes, natural killer (NK) cells, dendritic cells (DC)s, M1 macrophages, and suppressive immune cells such as myeloid-derived suppressor cells (MDSC)s, tumor-associated macrophages (TAM)s, regulatory T cells (Treg)s, etc. [15] (Figure 1). TILs in TME encompass stromal TILs, which are positioned in the tumor margins without direct contact with cancer cells and intratumoral TILs, defined as lymphocytes in direct cell-to-cell contact with tumor cells with no intervening stroma. Intratumoral TILs make the smaller proportion of TILs as endothelial cells and stromal tissue aggravate their infiltration into the tumor [16].

The abundance and the composition of TILs regarding the presence of distinct lymphocyte populations has been evaluated in multiple studies in the context of breast cancer disease prognosis and response to chemotherapy [16,17,18,19]. TNBC is characterized by a greater abundance of TILs compared to other molecular subtypes of breast cancer [20]. Moreover, in TNBC and HER2+ breast tumors, higher proportions of TILs have been associated with better response to chemotherapy. The same study indicated the presence of TILs as a favorable prognostic marker in early-stage TNBC [21]. Furthermore, the deficiency of stromal TILs and low number of CD8+ T cells were found to independently predict mortality in TNBC while the prognostic value of TILs and their CD8+ subset varied with respect to the cancer compartment [22]. In patients with TNBC distant metastases were shown to exhibit scarce presence of TILs compared to the primary tumor. The same study reported the longer median overall survival (OS) in the cohort of patients with metastatic lesions containing TILs>10% compared to patients with TILs<10% [23]. In distant metastases of TNBC, the presence of TILs identified patients with enhanced response to immunotherapy, suggesting the importance of immune activation for improving survival outcomes [24].

CD8+ T cells play an initial role in antitumor immune defense. The naïve T cells undergo priming by tumor-derived antigens presented by antigen-presenting cells (APCs) in regional lymph nodes, and migrate to TME via blood stream or lymphatics, where they differentiate into cytotoxic T cells (CTLs). CTLs directly kill tumor cells by producing cytotoxic enzymes perforin and granzymes. CD4+ T cells play an immunoregulatory role. Based on the cytokines they generate, CD4+ T cells are divided into Th1, Th2, Th17, and regulatory T (Treg) cell subsets, each of which has a unique function in immune responses [25]. Th1 T cells are the primary source of the inflammatory cytokines interferon (IFN)-ꝩ, interleukin (IL)-2, and tumor necrosis factor (TNF), which induce maturation of APCs, more effective presentation of tumor antigens, and maturation of CD8+ cells to fully functional CTLs. Conversely, Th2 cells secrete IL-4, IL-5, IL-6, IL-9, IL-10, and IL-13, which are responsible for activation of humoral immunity, eosinophil activation, inhibition of macrophage functions and suppression of antitumor immune responses [26]. Th17 cytokines IL-17, IL-21, and IL-22 are reported to facilitate cancer progression by promoting inflammation and angiogenesis. The elevated level of these cytokines in peripheral blood has been related to progression of breast cancer [27].

B cells in TME of breast cancer have been less studied in primary tumors compared to axillary lymph nodes. In antitumor immune response, B cells have a primary role in presenting antigens to CD8 T cells and secreting antibodies that induce tumor cell destruction via antibody-dependent cellular cytotoxicity (ADCC), as well as producing cytokines that further support antigen presentation by professional APCs. In invasive breast cancer, B cells in TIL population are associated with higher grade tumors and lymph node positivity, indicating the role of B cells in tumor aggressiveness [28]. There are opposing results regarding the association of B cells in TME with disease prognosis in TNBC. While several studies report the association of infiltrating B cell with larger tumor size, high histological grade, lymphovascular invasion, and lymph node metastases in early breast cancer [29], there are also data showing association of high densities of infiltrating B cells in primary tumors with a better response to neoadjuvant chemotherapy [30]. According to some research data, interactions between TNBC cells and B cells increase gene expression for a number of inflammatory cytokines, most notably IL-1β, which results in chronic inflammation [31]. IL-1β by activating nuclear factor-κB (NFκB), promotes angiogenesis and EMT. Furthermore, NFκB signaling was associated with large tumor size, high histological grade, and negativity for ER and PR expression in invasive breast cancer [32]. Newly defined subtype of B cells, the regulatory B cells (Bregs), have a role in immune tolerance, similar to regulatory T cells. In this sense, CD25+ and IL10+ B cells were recently related to the induction of Tregs in TIL aggregates and the development of metastasis in breast cancer [33].

NK cells are the subpopulation of innate lymphoid cells (ILCs) that have a unique ability to directly recognize malignantly transformed cells via cognate NK cell receptors and kill tumor cells by releasing cytotoxic enzymes perforin and granzymes. Aside from cytotoxic activity, which is performed without major histocompatibility complex (MHC) class I restriction, NK cells produce multiple cytokines—IFN-ꝩ, TNF, IL-10, IL-13—and granulocyte macrophage colony-stimulating factor (GM-CSF) that regulate immune responses [34]. NK cell antitumor activity is tightly regulated by the balance of signals transmitted by activating and inhibitory receptors bound to their ligands on tumor cells. NK cells are able to distinguish normal from transformed cells by killer cell immunoglobulin-like receptors (KIRs) that inhibit NK cell cytotoxic activity by binding to MHC class I molecules [35,36]. In tumor tissues, NK cells are mostly present during early tumorigenesis, but they become scarce in advanced stages of tumor development due to increasing immunosuppression in TME [37].

In this sense, several tumor evasion mechanisms overcoming NK cell-mediated tumor control have been identified, including the impaired NK cell recruitment to the tumor bed, shedding of ligands for NK cell activating receptors, and the induction of the inhibitory ligands on tumor cells [38,39]. The suppressive effect of TME on NK cytotoxic activity of NK cells can be mediated by cytokines such as TGF-β and IL-10, L-kynurenine, a product of tryptophan degradation, and prostglandin E2 (PGE2), secreted by tumor cells, suppressive immune cells, and CAFs [40]. These soluble factors in TME downregulate the expression of NK cell-activating receptors, including NK group 2D (NKG2D), NKp30, and DNAM1, as well as their signaling pathways [41,42,43]. Moreover, persistent stimulation of NKG2D receptor by its tumor cell ligands, as well as soluble ligands generated by proteolytic shedding mediated by matrix metalloproteinases (MMPs) produced by tumor cells or CAFs, may also lead to functional exhaustion of NK cells [42,44]. However, IFN-γ released during acute early inflammation in the context of antitumor immune response induces the resistance of tumor cells to NK cell-mediated lysis by upregulating classical and non-classical MHC class I molecules [45].

Cell-to-cell contacts in TME significantly affect NK cell functions. In this sense, ligand–receptor interaction between CAFs and NK cells was reported to inhibit NK cell cytotoxicity toward cancer cells, downregulate the expression of activating NK cell receptors, and thereby promote cancer cell escape from NK cell surveillance, according to a study that used a mouse model of breast cancer. Moreover, the tumor samples obtained from patients with TNBC showed enrichment of NK cells in CAF-rich regions, accompanied with upregulated binding of NK cell receptors to ligands on CAFs that altogether correlated with poor disease outcome [46].

Tumor infiltrating NK cells appear to have lower antitumor activity compared to circulating NK cells [47,48]. Moreover, in early stages of tumor development, NK cells exhibit antitumor activity, while in the later cancer stages they show impaired cytotoxic capacities and become senescent cells, as shown in the murine model of TNBC [49]. In one study in mice, a unique subcluster of Socs3highCD11b-CD27- of immature NK cells found specifically in TNBC samples showed reduced expression of cytotoxic granzyme signature and the ability to activate cancer stem cells through Wnt signaling. Furthermore, the same study reported increased numbers in functionally less mature CD56bright NK cell subset in tumor samples, which correlated with poor OS in patients with TNBC [50]. A recent study identified the distinctive IL-10+ secreting immunosuppressive NK cell population in TILs with a strong relation to poor survival prognosis in TNBC patients treated with neoadjuvant IC blockade immunotherapy [51].

### 2.1. Immune Checkpoint Receptors

IC receptors are upregulated as a consequence of immune responses, with their primary physiological role being the prevention of excessive immune reactions. IC receptor PD-1 and its ligand PD-L are important negative regulators of immune activity. During tumorigenesis, oncogenic pathways, genetic, and epigenetic factors intrinsic to tumor cells upregulate the expression of PD-L1 (B7H1) and PD-L2 (PD-L2) on tumor cells. In this sense, microRNA-200/ZEB1 axis, aberrant activation of Wnt signaling, loss of phosphatase and tensin homolog (PTEN), activation of phosphoinositide 3-kinase (PI3K)/protein kinase B (AKT)/mechanistic target of rapamycin (mTOR) signaling pathway, and MUC1-C/Myc/NF-Kβ axis were shown to upregulate the expression of PD-L1 in TNBC [52,53,54,55]. Inflammatory cytokines in TME, such as IFN-ꝩ, IL-1β, IL-6, and TNF, further favorize cancer immune escape by augmenting PD-L1/PD-L2 expression on tumor cells including TNBC [55,56]. Aside from tumor cells, multiple cells in TME, including immune cells (DCs, macrophages, Tregs) and CAFs, express PD-L1 which further contributes to suppressing antitumor immunity [57,58,59]. In this sense, in TME and tumor-draining lymph nodes, PD-L1 is often upregulated by IFN-ꝩ on APCs, leading to the inhibition of T cell activation [60].

The process of EMT is associated with augmentation of PD-L1 expression in multiple tumors. EMT-inducing transcription factors such as ZEB1 have been shown to bind to gene promoter region of PD-L1 gene [61]. By blocking the glycogen synthase kinase (GSK)3β-mediated phosphorylation, ubiquitination, and degradation of the EMT-inducing transcription factor Snail, PD-L1 has been shown to enhance EMT in TNBC cells, hence increasing the likelihood of metastasis [62].

Aside from PD-1 and CTLA-4, chronic inflammation in TME can result in upregulated expression of several other IC receptors contributing to immunosuppression and resistance of tumor cells to the antitumor activity of lymphocytes infiltrating the TME. In this sense, T cell immunoglobulin and mucin domain-containing protein 3 (TIM3), lymphocyte activation gene-3 (LAG-3), T cell immunoreceptor with immunoglobulin and ITIM domain (TIGIT), and V-domain Ig-containing suppressor of T cell activation (VISTA) are upregulated on T and NK cells [13]. Therefore, therapeutic targeting of multiple IC molecules on immune cells represents a challenge and a potential option for treatment of TNBC [reviewed in 10].

### 2.2. Suppressive Immune Cells in TME

#### 2.2.1. Regulatory T Cells

Regulatory T cells represent the functionally distinctive subpopulation of CD4+ T cells that express forkhead box P3 (FoxP3) transcription factor and high-affinity IL-2 receptor α chain (CD25), depleting IL-2 from the TME and thereby disturbing T cell proliferation and activation. Tregs play the key role in immunologic tolerance primarily by inhibiting the proliferation, cytotoxic activity, and cytokine production of T cells. Tregs suppress immune reactions by multiple mechanisms such as the production of immunosuppressive cytokines and other soluble factors, including TGF-β, IL-10, IL-35, and PGE2. Additionally, Tregs express IC molecules that altogether create an immunosuppressive environment by impairing antitumor activity of T and NK cells [63,64]. Breast cancer and stromal cells in TME cells secrete chemokines CCL2, CCL5 (RANTES), CCL20, CCL22, and CXCL12 which, by binding to the corresponding receptors, induce Treg homing to tumor tissue. It has been reported that by releasing CCR5-associated chemokines, mainly CCL3 (MIP-1α), CCL4 (MIP-1β), and CCL5, which are strong attractants for lymphocytes and macrophages, Tregs facilitate metastatic invasion of TNBC to lymph nodes and bone marrow [64,65,66].

Tregs constitutively and highly express IC receptor CTLA-4 and are able to down-regulate the expression of costimulatory molecules CD80/CD86 on APCs thereby hindering antitumor activity of T cells [67]. Inflammatory conditions in TME such as type I IFN signature, PD-L1, and expression of indoleamine 2,3-dioxygenase (IDO), which characterize immunomodulatory subtype of TNBC, contribute to acquisition of a strong immune-suppressive functional Treg phenotype [8,9]. Furthermore, it was reported that PD-1 in contact with PD-L1 could convert naive CD4+ T cells to Tregs through the downregulation of Akt, mTOR, and ERK2 and simultaneous upregulation of PTEN signaling pathways [68].

Although the presence of lymphocyte infiltrates seems to reflect favorable host antitumor immune responses, suggesting that immune activation is important for improving survival outcomes, the composition of lymphocyte infiltrate varies across molecular subtypes of breast cancer [69]. In this sense, the increased presence of Foxp3+ Treg subsets was reported in TNBC and HER-2-positive tumors compared to luminal subtypes [70]. Moreover, the same study indicated the association of greater Treg to CD8+ T cell ratios with higher grade tumors and higher Ki-67 expression. Changes in Treg infiltration were reported during the course of metastatic progression, indicating the relevance of Treg infiltration for unfavorable disease prognosis in breast cancer [71]. Furthermore, the presence of FOXP3+ lymphocytes in tumor tissues, along with TNM stage, has been identified as an independent prognostic factor for OS in TNBC [72]. Aside from their role in promoting metastatic invasion, high infiltration rate of Tregs in patients with TNBC has been associated with inhibited immune activation pathways and resistance to anti-PD1 immunotherapy [66].

#### 2.2.2. Tumor-Associated Macrophages

Tumor-associated macrophages (TAMs) may include macrophages of M1 antitumor phenotype that are present mostly in early phases of immune response to tumor and immunosuppressive M2 subset which is more abundant in TME of the growing tumor. M2 macrophages enhance immune tolerance, angiogenesis, tumor growth, and invasion. This subset of macrophages produces numerous anti-inflammatory factors (TGF-β, IL-4, IL-13, IL-10, IL-1RA), lower levels of inflammatory cytokines (IL-6, IL-12, IL-23, TNF-α), and considerable amounts of growth factors (EGF, vascular endothelial growth factor (VEGF)) [73]. Furthermore, by lowering the levels of arginine through the conversion of L-arginine to ornithine through arginase (Arg) activity, TAMs suppress T cell antitumor function [74]. The ornithine is further translated into polyamines (putrescine, spermidine, and spermine), which are linked to more immunosuppressive TME as they promote the growth and function of MDSCs, macrophages and Tregs, as well as collagen production in the process of tissue repair and survival of tumor cells [75,76]. Moreover, the expression level of Arg-1, which is one of the molecular markers of M2 macrophages, was elevated in peripheral blood, lymph nodes and tumor tissue of breast cancer patients [77]. Furthermore, TAMs produce multiple enzymes that participate in metastatic tumor invasion, such as MMPs, serine proteases, and cathepsins, and decompose collagen and other components of the ECM, thereby helping the migration of tumor and stromal cells [73]. There are also data indicating the role of M2 macrophages in EMT, as CCL2 produced by cancer and myeloid cells was shown to attract M2 macrophages and induce Wnt-1 upregulation. Wnt-1 in turn downregulates E-cadherin junctions in HER2+ early breast cancer [78]. Accordingly, in TNBC, the presence of M2 TAMs in TME was correlated with stromal fibroblast infiltration, EMT, and poor patient survival [79].

#### 2.2.3. MDSC

MDSCs are immature myeloid cells that arise after prolonged exposure of monocytes or neutrophils to inflammatory conditions in TME. Various tumor-derived factors induce differentiation of MDSCs in vitro, including PGE2, IL-6, IL-10, IL-1β, TGF-β, stem cell factor (SCF), and proangiogenic VEGF [80]. By expressing IC molecules and secreting immunosuppressive cytokines, most notably TGF-β, IL-1β, and IL-10, MDSCs suppress the activity of T and NK cells [81,82]. MDSCs also block T cell activation by sequestering cystine and limiting the availability of cysteine in TME [83]. Additionally, MDSCs contribute to tumor-induced immune suppression by blocking the production of IL-12 by TAMs and DCs that induces polarization of macrophages toward a tumor-promoting M2 phenotype. Furthermore, M2 macrophages play a role in the development and proliferation of FoxP3+ Treg cells [84].

Increased infiltration of MDSCs was reported in TNBC compared to other molecular subtypes of breast cancer [85]. The same study reported that the number of MDSCs population in patient with basal-like TNBC correlated with the level of transcription factor ΔNp63, which promotes tumor growth, progression, and metastasis in humans and in the mouse model of TNBC. The mechanism involved in MDSC recruitment was the ΔNp63-dependent activation of chemokines CXCL2 and CCL22. Furthermore, it has been revealed that MDSCs recruited into the TME of TNBC release the enzymes chitinase 3-like and MMP9, which are implicated in metastatic invasion and the promotion of cancer stem cell function [85,86].

#### 2.2.4. Tumor-Associated Neutrophils

Tumor mediated signals such as TGF-β promote the differentiation of a pro-tumorigenic N2 subtype of neutrophils. Tumor-associated neutrophils (TANs) produce high levels of TNF, inducible nitric oxide synthase (iNOS), NO, and H_2_O_2_, damaging DNA and inducing genetic instability. Therefore, N2 neutrophils represent active players in inflammation-related tumorigenesis and tumor development. Furthermore, this population of neutrophils supports tumor invasion, angiogenesis, and metastasis by secreting VEGFA and MMP9. Besides the secretion of soluble factors, TANs have been shown to impair the antitumor immune response by expressing immunosuppressive molecules, such as PD-L1. TANs are present in most TNBC tumors, indicating their potential to impact breast cancer prognosis [87].

## 3. Cancer-Associated Fibroblasts

Fibroblasts are mesenchymal cells with diverse physiological functions such as ECM synthesis and remodeling, tissue repair, mesenchymal lineage maintenance, secretion of signaling molecules, and immune regulation. Cancer-associated fibroblasts (CAFs) play a complex role in the TME because of their ability to influence many aspects of tumorigenesis, including the proliferation of malignant cells, motility, angiogenesis, metastatic invasion, inflammation, and metabolic reprogramming [88]. In breast cancer, up to 80% of normal fibroblasts in breast tissue acquire CAF phenotype during tumor progression [89,90].

Aside from “normal” fibroblasts, diverse cells of mesenchymal origin, such as adipocytes and mesenchymal stem cells, can give rise to CAF differentiation when exposed to the soluble factors in TME due to intrinsic intercellular plasticity. In this sense, bone marrow mesenchymal stem cells can be transformed into CAFs when exposed to conditioned medium in which tumor cells were cultivated [91]. Similarly, adipocytes and pericytes, as cells of mesenchymal origin, were reported to differentiate into fibroblasts when exposed to conditioned media from tumor cells [92,93]. Furthermore, in the proximity of the developing tumor and through the process of EMT triggered by soluble factor in TME, epithelial and endothelial cells can downregulate epithelial and endothelial markers, respectively, and acquire fibroblast-like characteristics [88].

Morphologically, CAFs are spindle-shaped mesenchymal cells characterized by overexpression of α-smooth muscle actin (α-SMA) that regulates cytoskeleton rearrangements contributing to cellular mobility. In breast cancer, the expression of α-SMA was reported to correlate with lymph node metastases and poor prognosis [89]. CAFs also express cytoskeletal protein vimentin, which is also a marker of EMT [94].

In tumor surroundings, growth factors, cytokines, various signaling molecules, metabolites released by cancer and immune cells, mechanical stress, alterations in histone acetylation, and DNA damage, all trigger differentiation of CAFs [95]. TGF-β plays a leading role in the generation of CAFs by activating canonical (SMAD-dependent) and noncanonical (non-SMAD) pathways [96,97]. In addition, TGFβ-1 secreted by activated fibroblasts (TGF-β RII) creates a positive feedback loop by binding to the type 2 TGF-β receptor, further increasing fibroblast activation. Aside from TGF-β, osteopontin (OPN), IL-1β and IL-6, secreted by cancer or immune cells, induce the conversion of stromal fibroblasts to CAFs by activating downstream TGF-β/Smads, NF-kB signaling, and signal transducer and activator of transcription (STAT)3 that are pivotal for modulating expression of genes linked to the CAF lineage [98]. Furthermore, metabolic reprograming towards aerobic glycolysis induced by lysophosphatidic acid (LPA), TGF-β1 or platelet-derived growth factor (PDGF) produced by tumor cells, activate hypoxia-inducible factor (HIF)-1α pathway [96] contributing to the differentiation of fibroblasts into CAFs. The central role in the establishment and maintenance of CAFs is associated with function of Yes-associated protein (YAP)1 and its paralog, transcriptional coactivator with a PDZ-binding motif (TAZ). In CAFs and tumor cells, the lack of YAP1 phosphorylation results in its translocation to the nucleus, its subsequent binding to TEAD transcription factors, and the activation of mitogenic pathways [99]. YAP regulates the expression of several cytoskeletal proteins. The activation of YAP occurs as a consequence of the ECM remodeling and stiffening induced by factors such as LPA and TGF-β. Src-family kinase function is required for YAP activity and expression of myosin light chain (MYL) 9, matrix stiffening, and many pro-tumorigenic properties of fibroblasts [100]. Moreover, cancer-derived exosomes induce fibroblast conversion to CAFs by shuttling not only microRNAs (miRNAs) and long noncoding RNA Gm26809, but also growth factors such as TGF-β1 [101]. Furthermore, PDGF drives pericyte differentiation into fibroblasts [90,92].

Aside from previously mentioned cytoskeletal markers, CAFs were also found to express fibroblast activation protein (FAP), CD29 (integrin β1), PDGF receptors α and β (PDGFRα and PDGFRβ), CD90 (THY-1), podoplanin (PDPN), fibroblast-specific protein 1 (FSP-1, S100A4), and caveolin 1. However, these markers are neither uniquely expressed nor necessarily coexpressed in CAFs [102].

The FAP serine protease is exclusively present in the TME and absent in healthy tissues. Its function is to enzymatically remodel the ECM, thereby enabling cellular migration. In HER2+ and TNBC, this tumor-promoting protein is enriched in CAFs expressing PD-L1/2 which, by binding to PD-1, have a potential to inhibit T cell activity [102]. Based on a recent study comprising integrated analysis of single-cell RNA sequencing data, clinical specimens, in vivo, and in vitro experiments, FAP+ CAFs have been identified as the predominant stromal population associated with poor clinical outcomes and immunosuppressive features in breast cancer patients. FAP+ subset of CAFs by secreting high levels of fibronectin 1 which binds to integrin α5β1 on macrophages was found to triggers macrophage polarization toward immunosuppressive M2-like phenotype by activating focal adhesion kinase (FAK)-AKT-STAT3 signaling [103].

The CD90 glycoprotein plays a major role in cell migration, adhesion, and fibrosis by regulating interactions between cells or between cells and the ECM. High expression of CD90 in breast cancer CAFs was associated with increased cell transformation and unfavorable disease prognosis, particularly in the basal-like TNBC subtypes [104].

The high expression of integrin α11/PDGFRβ on stromal cells was associated with high tumor grades and unfavorable clinical outcome in breast cancer patients. Pro-invasive function of integrin α11 relies on its ability to interact with PDGFRβ that promotes downstream JNK activation, leading to the production of pro-invasive ECM protein tenascin C [105].

FSP-1, the small calcium-binding integral membrane protein, is a serine protease often expressed in CAFs in primary breast cancer and in matching lymph node metastasis as well as in macrophages, other immune cells, and cancer cells. FSP-1+ CAFs produce the ECM component tenascin C, cytokines, MMPs, and VEGF-A, thereby facilitating cell motility, angiogenesis and metastatic invasion [106]. Moreover, the expression of FSP-1 was associated with unfavorable prognosis and may be considered a prognostic indicator of metastatic disease in early breast cancer [107,108].

Pro-tumorigenic CAFs in breast cancer often express CD10, a Zn-dependent matrix metalloproteinase which was predominantly related to ER-negative invasive breast cancer, whereas CD10- CAFs were associated with luminal type invasive breast cancer [109]. GPR77, the non-G protein-coupled complement receptor, when expressed in combination with CD10, defines distinct CAF subpopulation that was related to chemotherapy resistance and poor prognosis in patients with lung and breast cancer. CD10+GPR77+ CAFs supply the TME with IL-8 and IL-6, which through the activation of NF-κB signaling via p65 phosphorylation and acetylation, protect cancer stem cells from chemotherapy-induced cell death [110,111,112].

However, the low expression of some markers, such as caveolin 1 scaffold protein, in CAFs is related to a more advanced tumor stage, early cancer recurrence, lymph node metastasis, and poor disease prognosis in breast cancer [113].

### 3.1. Subpopulations of Fibroblasts in TNBC

CAFs are a heterogeneous population with respect to their embryonic and spatial origin and functional characteristics. Several attempts have been made to classify CAFs in breast cancer according to the expression of molecular markers and functional properties. In this sense, single-cell RNA sequencing and histological characterization performed on breast cancer mouse models have defined three distinct CAF compartments including the peri-vascular niche, the mammary fat pad, and the transformed epithelium, with each CAF subset being clearly distinctive for the expression of specific gene programs [114,115,116].

According to one study in breast cancer patients, based on the expression of specific markers CD29, FAP, FSP1, α-SMA, CAV-1 and dipeptidylpeptidase 4 (DPP-4, CD26), CAFs can be classified into four subtypes: CAFs-S1 to S4 [102]. CAFs-S1 (CD29Med FAPHiFSP1Med αSMAHigh PDGFRbMed-High CAV1Low) and CAF-S4 (CD29High FAPNeg FSP1Low-Med αSMAHigh PDGFRbLow-Med CAV1Low) subsets were detected preferentially in TNBC and HER-2-negative breast cancer. CAF-S1 fibroblasts were associated with immunosuppression in the TME by secreting CXCL12, which attracts CD4+CD25+ T lymphocytes and retains them by T cell-interacting proteins, namely OX40L, PD-L2, and JAM2. Moreover, CAF-S1 has been shown to promote T cell differentiation into CD25HighFoxP3High Tregs, through the involvement of B7H3, CD73, and DPP4 [102]. Recent studies led to the establishment of gene profiles of CAF subtypes that were associated with distinctive functional programs and indicated some prognostic significance in clinical settings. In this sense, the CAF-S1 and CAF-S4 subsets were reported to accumulate in LN and correlate with cancer cell invasion while the CAF-S2 and CAF-S3 subsets share similar gene expression profile with fibroblasts found in normal breast tissue. The same study reported that CAF-S1 stimulates cancer cell migration and initiates the EMT through CXCL12 and TGF-β pathways. However, according to this study, the highly contractile CAF-S4 induce cancer cell invasion via Notch signaling. Moreover, the patients with CAF-S4 in LN at diagnosis were prone to develop late distant metastases [117].

In their study, Friedman et al. used index and transcriptional single-cell sorting during breast cancer progression in mice and identified eight CAF subtypes, grouped into two main CAF populations, pCAF and sCAF, based on selective expression of the markers PDPN or S100a4 (FSP1). These CAF subtypes appeared progressively over time, transitioning from an early immune-regulatory transcriptional program to the combination of antigen-presentation and wound-healing programs. The same study reported that human breast tumors have similar CAF compositions and that the ratio between PDPN+ and S100A4+ CAFs was in TNBC associated with BRCA mutations. Furthermore, patients with BRCA1/2 mutations exhibited significantly lower PDPN staining, and higher S100A4/PDPN ratios compared to patients with BRCA wild-type tumors [118].

Another study involving single-cell RNA sequencing of TNBCs specimens of five patients defined four subpopulations of CAFs based on the expression of gene programs, morphology, spatial relationships and functional properties [115]. In this context, myofibroblast-like CAFs showed the features of myofibroblasts while the inflammatory-like CAFs (iCAFs) were characterized by high expression of growth factors and immunomodulatory molecules. The two remaining subsets were designated as differentiated or immature perivascular-like cells. According to this study, iCAFs and differentiated perivascular-like cells in a cohort of TNBC patient revealed strong association with cytotoxic T cell-dysfunction and T cell-exclusion gene signatures, respectively [116].

In conclusion, a better definition of molecular markers for distinct CAF populations may aid in development of novel therapeutic approach based on biomarker-oriented therapeutic targeting of CAFs.

### 3.2. Immunosuppressive Factors Secreted by CAFs

**Figure 2:**
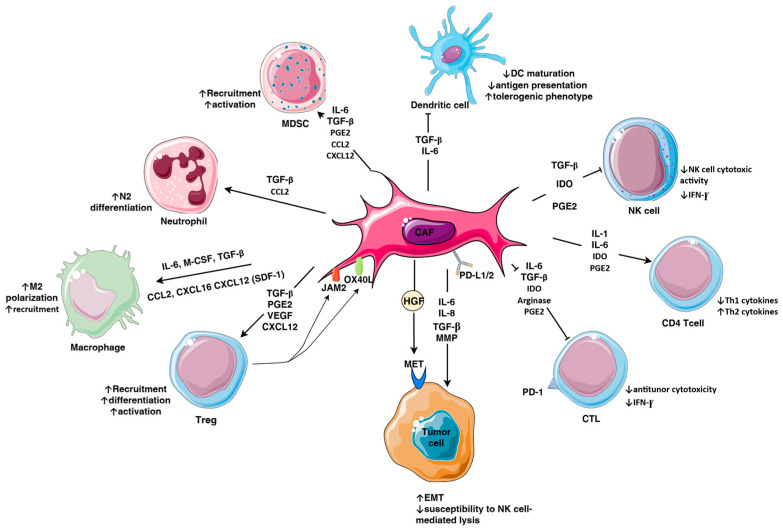
Immunomodulatory mechanisms exerted by cancer-associated fibroblasts (CAF) in triple-negative breast cancer (TNBC). By secreting multiple cytokines, chemokines, growth factors, and enzymes, CAFs suppress antitumor immune responses through several mechanisms such as hindering presentation of antigens by dendritic cells (DC), inhibiting antitumor activity of cytotoxic T (CTL) and natural killer (NK) cells, and suppressing production of Th1cytokines. Furthermore, CAFs promote differentiation and polarization of suppressive immune cells, namely, myeloid-derived suppressive cells (MDSC), M2 subset of macrophages, regulatory T cells (Treg), and N2 subset of neutrophils, and induce their recruitment to tumor microenvironment (TME). By modulating phenotype of tumor cells through secretion of matrix metalloproteinases (MMPs) that induce shedding of ligands for activating NK cell receptors (NKG2D, DNAM1) thereby reducing tumor cell susceptibility to NK cell-mediated lysis, induction of epithelial to mesenchymal transition (EMT) of tumor cells, activation of signaling pathways such as MET tyrosine kinase, CAFs contribute to immune evasion in TME. Furthermore, direct cell–cell contacts between CAFs and CTLs such as PD-L1 to PD-1 and CAF retention and attraction of Tregs through OX40L, PD-L2, and JAM2 contribute to suppression of antitumor immune response in TNBC.

CAFs modulate TME by secreting multiple cytokines, growth factors, chemokines, enzymes, and metabolites that contribute to tumor progression, metastatic invasion, and resistance to chemo- and immune therapy of TNBC (Figure 2).

In this sense TGF-β secreted by CAFs induces expression of EMT-related transcription factors (TWIST, Snail), cytoskeletal components (fibronectin, vimentin), and MMP-2/9, that altogether increase cancer cell motility and facilitate metastatic invasion. Through the Smad pathway, TGF-β increases the expression of high-mobility group A2 (HMGA2) protein which regulates the expression of repressors that downregulate the transcription of E-cadherin during the EMT of tumor cells [119]. Inhibitory effects of TGF-β on antitumor immune responses, such as differentiation of suppressive immune cells, downregulation of activating receptors on NK and T cells, and impairment of T and NK cell antitumor functions, have been well described in multiple studies [120]. CAFs inhibit the functions of effector T cells by secreting IL-6, TGF-β and chemokines (CXCL9, CXCL12) and directly by cell-to-cell interactions such as PD-1/PD-L1 receptor signaling [121,122]. In support of receptor–ligand mediated suppression of T cell activity, the integration of spatial and single-cell transcriptomics revealed that IGF2 promoted interactions between CAFs and T cells via CXCL12 and PD-L1 [123].

CAFs contribute to differentiation of suppressive immune cell subsets. In this sense, by secreting IL-6 and TGF-β, CAFs downregulate the expression of costimulatory (CD49, CD80) and antigen-presenting MHC class II molecules, thereby hindering the functional maturation of DCs and consequently impairing the appropriate antigen presentation. In this manner DCs differentiate into regulatory DCs that express high levels of IDO1, depriving TME of tryptophan by converting it to kynurenine and thus suppressing the proliferation of T cells and supporting the differentiation of Tregs [124,125]. By producing monocyte chemoattractant proteins CCL2 and CXCL16, iCAFs facilitate the recruitment of monocytes into TME and M2 polarization [126]. Accordingly, it was reported that CAF-secreted CXCL16 draws monocytes and creates a migratory feedback loop that intensifies the development of a reactive stroma and facilitates the invasiveness of TNBC tumors [127]. Moreover, the clinical data showed association of CAFs with infiltration of macrophages and tumor invasion into lymph nodes [128]. Furthermore, it was reported that CAFs contribute to immunosuppressive PD-1+ M2 phenotype and increased production of anti-inflammatory cytokine IL-10 by monocytes recruited into breast tumor [126].

Furthermore, CAFs inhibit the production of Th1 cytokines, cytotoxic activity and proliferation of CD8+T and NK cells by producing PGE2, while they induce the secretion of Th2 cytokines and stimulate the expansion of Tregs [129]. CAFs positive for sialoglycoprotein podoplanin (PDPN+ CAFs) were reported to produce high levels of immunosuppression-promoting enzymes IDO1 and tryptophan 2,3-dioxygenase (TDO)2. Moreover, PDPN+ CAFs were detected in breast tumors of patients unresponsive to the treatment with trastuzumab (anti-HER2 monoclonal antibody) [130].

One of the major contributions of CAFs to immunosuppression in the TME is through the secretion of chemokine stromal-derived factor-1α (SDF1, CXCL12) that by binding to its G-protein-coupled CXCR4 receptor on MDSCs, Tregs, and monocytes, induces their recruitment into the TME. The microenvironment of TNBC showed increased expression of CXCL12 and its CXCR4 receptor [131]. In response to tissue damage and inflammation in TME, CAFs attract polymorphonuclear leukocytes by secreting CXCL12, which in the presence of tumor-derived CCL20, additionally upregulate PD-L1 expression in neutrophils consequently inhibiting antitumor activity of T cells [132,133]. Furthermore, CXCL12 by binding to its receptor on nearby stem cells (CD44+CD24-) accelerates the development of cancer by encouraging proliferation and malignant initiation [134,135]. Recent studies have been focused on the development of CXCR4 receptor antagonist to alleviate immune suppression in TME and improve the outcome treatment of IC inhibitors in TNBC [136].

Additionally, CAFs secrete hepatocyte growth factor (HGF) and activate MET receptor tyrosine kinase which is frequently overexpressed in TNBC cells. This tumor–stromal interaction promotes invasiveness, EMT, and activation of multiple oncogenic pathways in TNBC cells [137,138].

### 3.3. ECM Remodeling

In the TME, aside from suppressing antitumor immunity, CAFs produce collagen types I, III, IV, and V, plasmin, hyaluronic acid, and laminin components of the ECM [139]. This is designated as tumor desmoplasia, a cancer-specific type of fibrosis, which is induced by ECM remodeling occurring during genesis of the majority of solid tumors. CAFs facilitate focal adhesion formation and alignment of the ECM proteins into parallel fibers, directing the local invasion of tumor cells. This is accompanied by the enzymatic degradation of ECM by MMPs and urokinase-plasminogen activators. Type I collagen is the most abundant component of ECM in breast cancer that plays an important role in supporting the survival and proliferation of tumor cells. Moreover, it has been reported that type I collagen, secreted by stromal fibroblasts, contributes to metastatic invasion of breast cancer cells through the induction of mRNA for MMP-9 [140,141,142]. Infiltrating immune cells, such as TAMs and TANs, play an active role in the activation of CAFs to produce interstitial ECM components by secreting cytokines and growth factors and stimulating oncogenic pathways such as Wnt/catenin-β signaling [93]. Furthermore, lysyl oxidase (LOX) produced primarily by TAMs and CAFs catalyzes oxidation of lysine residues, inducing collagen cross-linking and resulting in the reinforcement of the ECM by turning it into a defense and a stiffened structure [143].

The deposition of fibronectin by the CAFs intensifies intracellular tension via increased interaction with actin filaments. The high levels of fibronectin and its splice variants, cross-linked collagen I, and tenascin-C were associated with poorer survival or time to progression in breast cancer patients [140,144,145]. Additionally, ECM components, such as hyaluronan produced by both tumor cells and stromal cells, absorb water, causing the ECM to swell and elevate interstitial pressure.

In solid tumors, dense ECM with elevated interstitial pressure leads to chronic hypoxia. The hypoxic conditions contribute to the production of VEGF and PDGF by CAFs, inducing the migration and proliferation of endothelial cells and subsequent tumor neovascularization. This was evidenced in breast cancer via HIF-1α interaction with G protein-coupled ER (GPER) signaling pathway [146]. Together with hypoxic conditions, TGF-β and IL-6 were shown to induce VEGFA secretion by CAFs and breast cancer cells, thereby contributing to angiogenesis [147,148].

Through modulation of the ECM, CAFs foster interactions between tumor cells and soluble mediators in TME, i.e., cytokines, chemokines, etc., enhance tumor cell migration, subsequent metastatic invasion, and chronic hypoxia. Furthermore, ECM stiffness can affect cancer cell sensitivity to chemotherapy and molecular targeted drugs. The apparent example of this was reported for anti -HER2 therapy in breast cancer, revealing that the high collagen content in HER2+ tumors correlated with resistance to HER2-targeted therapies [144].

### 3.4. CAFs and Tumor Metabolism

According to the concept of reverse Warburg effect, tumor cells form metabolic symbiosis with CAFs in which cancer cells induce aerobic glycolysis and fermentation in neighboring stromal fibroblasts through induction of oxidative stress and TGF-β secretion. Subsequently, CAFs secrete lactate and pyruvate which cancer cells use in the mitochondrial tricarboxylic acid cycle, thereby promoting efficient energy production and ATP generation via oxidative phosphorylation. This results in a higher proliferative capacity of tumor cells. By these means, tumor cells modify the normal stroma according to the needs of growing tumor for energy-rich metabolites [149]. In patients with TNBC, the increased levels of monocarboxylate transporter-(MNCT)4 involved in the proton-dependent transport of L-lactate, pyruvate, and short chain fatty acids were associated with unfavorable prognosis [150]. Furthermore, exosomes secreted by breast cancer cells were found to activate the c-Myc protooncogene in CAFs, causing them to release exosomal factors that increase glutamine-dependent reductive carboxylation and glycolysis in cancer cells while inhibiting mitochondrial oxidative phosphorylation [151]. CAFs are also involved in metabolism of glutamine which, through its conversion to α-ketoglutarate, enters Krebs cycle and represents the second most important source of energy for tumor cells [152]. In hypoxic conditions CAFs secrete abundant colony-stimulating factor (CSF)3 that was reported to promote the invasive behavior of TNBC cells by activating its receptor, CSF3R. Subsequently, by activating its downstream target phosphoglucomutase 2-like 1 (PGM2L1), CSF3R enhances glycolysis, thereby providing energy to support the malignant phenotype of breast cancer. In vivo studies have further confirmed these findings in the context of TNBC progression [153].

In summary, CAFs contribute to the progression of TNBC by secreting growth factors and enzymes, producing increased amounts of ECM, and suppressing immune responses against tumor cells. Moreover, by creating an immunosuppressive environment and favorizing desmoplastic reaction that elevates interstitial pressure resulting in chronic hypoxia, CAFs confer resistance of cancer cells to standard therapies.

## 4. Cancer-Associated Adipocytes

Adipocytes represent a major stromal cell population in the breast tissue. Cancer-associated adipocytes (CAAs) are a distinct subpopulation of adipocytes present in the proximity of cancer cells at the invasive front of the breast tumor [154]. Wnt ligands and exosomes produced by breast cancer cells block the peroxisome proliferator activated receptor (PPAR)γ nuclear receptor, causing “normal” adipocytes to dedifferentiate and generate CAAs [155,156]. CAAs differ from “normal” adipocytes as they display fibroblast-like morphology, usually contain several dispersed small lipid droplets, and produce increased amounts of inflammatory cytokines IL-1β, IL-6, IL-8, TNF, chemokines (CCL2, CCL5), and leptin, which altogether facilitate tumor progression. Moreover, CAAs release free fatty acids (FFAs) which are transferred into tumor cells via CD36 scavenger receptor and undergo β-oxidation, subsequently delivering energy and building blocks to rapidly proliferating cancer cells [157]. CAAs also release other metabolites including lactate, ketone bodies, pyruvate, glutamine, and arginine, to meet the metabolic needs of tumor cells [158]. CAAs show increased expression of fibroblast-like biomarkers, such as α-SMA, FAP, chondroitin sulfate proteoglycan, and decreased expression of PPAR and fatty acid-binding protein. By producing inflammatory cytokines, VEGF, and IGF-1, CAA promote proliferation, EMT, migration, angiogenesis, and metastatic invasion of tumor cells. Furthermore, CAAs contribute to immunosuppression in the TME by downregulating antitumor activity of CTLs and NK cells, inducing recruitment and M2 polarization of macrophages [154]. By secreting adipokines and cytokines, CAAs participate in the remodeling of the ECM in breast cancer by regulating synthesis of fibronectin, MMPs, as well as synthesis and crosslinking of collagen [156,159]. CAAs secrete and process collagen VI, which activates NG2/chondroitin sulfate proteoglycan receptors on the surface of malignant ductal epithelial cells to sequentially activate Akt and β-catenin signaling and stabilize cyclin D1. This mechanism promotes the invasive behavior of breast tumor cells [160]. Adipocytes not only function as energy storage cells by providing FFAs for fueling cancer cells, but also serve as endocrine cells by secreting aromatase, the rate-limiting enzyme in estrogen biosynthesis which promotes the growth and progression of ER+ breast cancer [161].

## 5. Therapies Targeting TME in TNBC

In recent years, strategies to therapeutically target TME have emerged as a promising approach for cancer treatment due to the critical roles of the TME in tumor progression and modulation of response to standard-of-care therapies. This approach has the utmost relevance in the context of TNBC considering the low response rate and low duration of response to standard of care chemotherapy and IC inhibitor therapy. In this sense, there are multiple components in TME that appear as potential therapeutic targets.

### 5.1. Targeting Cytokines, Enzymes, Metabolites, Chemokines, and Immune Cell Recruitment

Being an omnipresent tumor-promoting factor in TME, TGF-β has been evaluated as therapeutic target in multiple studies in solid tumors [162]. In this sense, two antibodies targeting TGF-β type I receptor (RI), also known as the activin receptor-like kinase (ALK)5, the PF-06952229 and vactosertib (TEW-7197), are currently available. The phase I clinical study (NCT03685591) showed that PF-06952229 had good tolerability, manageable toxicity, durable responses, and/or disease stabilization in a small group of patients (Table 1). The study included dose escalation for monotherapy and combination therapy with cyclin-dependent kinases 4 and 6 (CDK4/6) inhibitor used in the therapy of metastatic ER-positive and HER2-negative breast cancer [163]. Vactosertib demonstrated considerable antitumor activity in a phase I clinical study (NCT02160106) in subjects with advanced-stage solid tumors including breast cancer [164]. However, further clinical investigations have shown the increased benefit of anti-TGF-β therapy in combination with other therapeutic modalities. In this sense, PF-03446962, as another monoclonal antibody developed against ALK1 of the TGF-β RI, failed to induce significant antitumor effects in breast cancer patients as a monotherapy according to the results of phase I clinical study (NCT00557856), while in combination with either bevacizumab or VEGFR tyrosine kinase inhibitor, it showed a greater reduction in tumor growth [165]. Furthermore, the next-generation TGF-β RI small-molecule inhibitor LY3200882 is currently being evaluated for safety in a phase I clinical study (NCT02937272) in patients with diverse solid tumors. The same clinical study showed good safety and tolerability for LY3200882 in combination with chemotherapy and anti-PD-L1 agent (LY3300054) [125,166].

Most notably, anti-TGF-β therapy has been used to improve the effect of IC inhibitors. In this sense, clinical phase Ib study (NCT02423343) investigating the combination of anti-PD-1 therapy (nivolumab) with novel TGF-β RI kinase inhibitor galunisertib monohydrate (LY2157299) in advanced solid tumors has been completed and showed good tolerability [167].

In the past decade, bifunctional antibodies targeting IC and TGF-β signaling have been developed. In this sense, bifunctional antibody SHR-1701 directed against extracellular domain of TGF-β RII and PD-L1 is currently being investigated in a phase I study (NCT03710265) in subjects with metastatic or locally advanced solid tumors to assess the safety and tolerability of different dose levels [168].

Furthermore, bintrafusp alfa, another bifunctional antibody targeting TGF-β RII and PD-L1 in phase Ib/II clinical (NCT03579472) study in patients with metastatic TNBC, was investigated in combination with eribulin mesylate. The study showed that the agent was generally well tolerated and showed promising antitumor activity [169].

HCW9218, a bifunctional antibody combining simultaneous TGF-β-neutralizing and immune-stimulating properties, was designed to target extracellular domains of human TGF-β RII and the IL-15 receptor α. HCW9218 is presently being investigated in a phase I first in-human clinical trial (NCT05322408) to determine the maximum tolerated dose in advanced or metastatic solid tumors. Furthermore, the study in mice treated with HCW9218 in combination with anti-PD-1 therapy demonstrated modulation of immune landscape in tumor-draining lymph nodes and enhanced T cell antitumor activity [170].

#### 5.1.1. Targeting IL-6

Targeting IL-6/JAK/STAT-3 pathway is another therapeutic approach directed against the TME, as IL-6 regulates many tumor-promoting functions. According to previous studies, TNBC cells secrete autocrine IL-6 and are less responsive to paracrine IL-6 signaling. The exposure to IL-6 leads to the chronic induction of STAT3 phosphorylation, which promotes further growth and invasion of these tumor cells [171]. Monoclonal antibody against IL-6 receptor, tocilizumab (TCZ), inhibited in vivo growth of MDA-MB-231 tumor cells and reduced the formation of metastasis in mice. Furthermore, phase I and II clinical trials demonstrated the efficacy of monoclonal antibodies against IL-6 and its receptor, either as single agents or in combination with other chemotherapeutic agents, radiation, and targeted therapies in various types of cancer [172]. In recent years the clinical use of TCZ was mainly restricted to mitigation of immune-related adverse events in patients treated with IC inhibitors and showed promising results in phase II clinical study (NCT04375228) [173].

The inhibition of the STAT3 signaling represents another experimental therapeutic strategy. In this sense, OPB-51602, the small-molecule inhibitor of STAT3 phosphorylation was evaluated in phase I (NCT01423903) multiple dose escalation clinical trial to determine safety and tolerability in subjects with advanced cancers for whom there is no standard treatment available. Furthermore, STAT3 inhibitor VVD-130850 is currently being investigated in a phase I clinical study (NCT06188208), both as a single agent and in combination with IC inhibition to evaluate its safety, tolerability, and preliminary antitumor activity in participants with advanced solid and hematological tumors [174].

Additionally, the polykinase inhibitor IMX-110 targeting STAT3 in combination with anti-PD1 agent tislelizumab is currently being investigated a phase I/IIa in dose escalation/dose expansion clinical study (NCT05840835) designed to assess its safety, tolerability, pharmacokinetics and antitumor activity in patients with advanced solid tumors [125].

The next-generation antisense oligonucleotide inhibitors of STAT3, AZD9150, and AZD5069, are currently being evaluated in combination with MEDI4736 (durvalumab), in a phase I clinical study (NCT02499328) in advanced solid malignancies to compare the effects of STAT3 inhibition monotherapy and its combination with anti-PD-L1 therapy. A similar phase I clinical study (NCT03394144) conducted on Japanese adult patients with advanced solid malignancies showed good tolerability for monotherapy and combination of this agent with durvalumab [175]. However, comparing the number of STAT3 inhibitors developed, only a small fraction is currently in clinical trials, perhaps due to the severe toxicities of the most of them [176].

#### 5.1.2. Targeting IL-8

Due to its role as a chemokine that promotes EMT of tumor cells, the recruitment of MDSCs to tumor site, subsequent immune escape, and its frequent overexpression in malignancies, including TNBC, IL-8 has been evaluated as a therapeutic target. Preclinical data showed therapeutic potential for clinical-stage monoclonal antibody that neutralizes IL-8 (HuMax-IL8) as it was shown to revert EMT in claudin-low TNBC models. The same study showed a significant decrease in the recruitment of MDSCs to tumor sites, an effect substantiated in treatment combination with docetaxel in TNBC [171,177]. Anti-IL-8 monoclonal antibody HuMax-IL8 (BMS-986253) was evaluated in phase Ib clinical study (NCT02536469) and showed good safety and tolerability, while the ongoing studies are presently evaluating the combination of IL-8 blockade and IC inhibitors in melanoma [178].

#### 5.1.3. CXCR4 Antagonist

A potent selective CXCR4 antagonist, the bilixafortide (B)- POL6326 is a synthetic cyclic peptide. POL6326 showed encouraging safety/efficacy data in phase I clinical trial (NCT01837095) in combination with eribulin, the synthetic analog of a natural product that inhibits microtubule dynamics and induces apoptosis of cancer cells, in second line treatment options of patients with relapsed TNBC and hormone refractory ER-positive metastatic breast cancer [179]. The preliminary activity of the combination showed promising results in patients with HER-negative metastatic breast cancer that led to phase III clinical trial (NCT03786094) in locally recurrent or metastatic breast cancer.

#### 5.1.4. IDO1 Inhibition

IDO1 allows tumor escape through kynurenine production which stimulates Treg activity while suppressing the proliferation of effector T cells. Preclinical findings have revealed that IC inhibitors, while removing molecular brakes on cytotoxic immune cells, also stimulate the production of IDO1 in TME, thereby activating a negative feedback loop in immune responses. Based on this, pharmacological IDO1 inhibitors are currently being tested in clinical studies (Table 2), mostly in combination with IC inhibitors [180]. In this sense, phase I clinical study (NCT02658890) evaluating BMS-986205 (Linrodostat) IDO-1 inhibitor, in combination with nivolumab alone, and both nivolumab and ipilimumab (anti-CTLA-4) (NCT02658890), showed favorable safety and efficacy in heavily pretreated patients with advanced solid tumors [181]. Furthermore, two clinical studies investigated the possibility of using BMS-986205 IDO1 inhibitor in combination with LAG-3 inhibitor relatlimab, CTLA-4 inhibitor ipilimumab, and PD-1 inhibitors in advanced and metastatic solid tumors (NCT03335540; NCT03459222), concluding that this therapeutic approach should be further investigated [182].

Epacadostat represents another IDO1 inhibitor that competes with Trp for binding to the catalytic site of the enzyme. Recently, epacadostat was evaluated in addition to chemotherapy and pembrolizumab in phase I/II clinical study (NCT03085914) and showed acceptable safety profile and antitumor activity across multiple types of advanced or metastatic solid tumors [183]. However, a phase Ia/1b clinical study (NCT03343613), investigating an anti-IDO1 agent LY3381916 administered alone or in combination with PD-L1 inhibitor LY3300054 in advanced solid tumors including TNBC, was terminated without a conclusion [184].

#### 5.1.5. Targeting PGE2

PGE2, another potential therapeutic target in TME, mediates its activity mainly through its G protein-coupled receptors EP2 and EP4 [185]. PGE2 suppresses the immune response by inhibiting the function of T cells, NK cells, and M1 macrophages, thus promoting tissue regeneration and inducing the differentiation of Tregs. In vivo studies have shown that the administration of EP4 antagonists restored the cytotoxic activity of NK cells in the context of progressive tumor growth [186]. Preclinical studies in breast cancer have shown NK cell-dependent antimetastatic activity of two PGE2 inhibitors: frondoside-A, the antagonist of EP4/EP2 receptors derived from the sea cucumber, and RQ-15986, the newly synthetized antagonist of the EP4 receptor [186,187]. Further studies led to the development of several antagonists for the EP4 receptor for clinical use. In this sense, the first in-human phase I study of E7046, a highly selective small-molecule antagonist, showed manageable tolerability in patients with advanced malignancies, immunomodulatory effects, the best clinical response of stable disease, and recommended the dose for phase II clinical investigation (Table 3) [188]. Phase I/IIa clinical trial (NCT05944237) is currently investigating the best dose of HTL0039732, a potent small-molecule antagonist of EP4, for its sole application in metastatic solid tumors and its joint use with atezolizumab [189]. Furthermore, a novel EP4 antagonist AN0025 was investigated in combination with pan-class I PI3K inhibitor buparlisib (AN2025) and atezolizumab in a phase Ia clinical study (NCT04975958) in patients with advanced solid tumors [190].

#### 5.1.6. PI3Kγ Inhibition

In preclinical studies, a selective inhibitor of PI3K-γ, eganelisib (IPI-549), showed potent ability to reshape the TME by reducing myeloid cell recruitment to tumors and reprogramming TAMs from immune-suppressive to immune-activating phenotype. This effect enhanced the activity of anti-PD-1/PD-L1 therapy [191]. Two complementary clinical studies have investigated eganelisib in combination with IC inhibitors. In this sense, eganelisib alone, and in its combination with nivolumab, was evaluated for safety and tolerability in a phase I/Ib clinical study (NCT02637531) in patients with advanced solid tumors. Simultaneously, the phase II MARIO-3 clinical study (NCT03961698) investigated the inhibition of PI3K-ꝩ, in combination with tecentriq (atezolizumab) and abraxane (nab-paclitaxel) in front-line TNBC (Table 4). The MARIO-3 study revealed elevated gene signatures of TAM reprogramming and immune activation in patients with TNBC with longer progression-free survival, regardless of the baseline PD-L1 status [192].

### 5.2. CAF-Directed Therapies

Based on recently defined roles of CAFs in immune suppression and resistance to therapy, direct targeting of CAFs and their surface markers was established as annovel and attractive target for anticancer therapies in advanced breast cancer (Table 5) [102,110].

RO7300490 represents a bifunctional antibody (Table 3) with potential immunostimulatory and antineoplastic activity. RO7300490 is a CD40 agonist that also targets the inducible tumor stromal antigen FAP-α protease, which is broadly expressed in CAFs in various solid tumors. RO7300490 displays its immunostimulatory effect by activating CD40 cell surface stimulatory TNF family receptor expressed on immune cells. CD40 engagement induces the proliferation and activation of B lymphocytes, differentiation of immunostimulatory macrophages, and activation of DCs to secrete inflammatory cytokines. This promotes the proliferation and activation of CTLs to kill tumor cells. A phase I clinical study (NCT04857138) evaluating safety, pharmacokinetics, and antitumor activity of RO7300490, as a single agent or in combination with atezolizumab, in participants with advanced and/or metastatic solid tumors, showed a favorable safety profile, and supported further clinical investigations of this agent in combination with other anticancer therapies [193].

Preclinical studies have consistently implicated FGFR signaling in breast cancer progression, while clinical evidence failed to support these findings. This may indicate that the clinical significance of FGFR should be analyzed in the context of the stroma, as the activation of resident fibroblasts can be a crucial factor for CAF generation and tumor progression [194]. Erdafitinib, a tyrosine kinase inhibitor of FGFR, was investigated in a phase Ib clinical study (NCT03238196) in combination with fulvestrant and palbociclib in ER+/HER2-/FGFR-amplified metastatic breast cancer. The study suggested some antitumor efficacy of this combination. Further investigations of erdafitinib in phase II RAGNAR (NCT04083976) clinical trial showed clinical benefit in tumor-agnostic setting in patients with advanced solid malignancies with susceptible FGFR alterations who have exhausted other treatment options. The results of this trial support the continued development of pharmacological inhibitors of FGFR for patients with advanced solid tumors [195].

The hedgehog signaling pathway is activated in the stroma of TNBC in response to hedgehog ligand secreted by cancer epithelial cells. Accordingly, a therapeutic approach that combines targeting cancer epithelial cells with chemotherapy and CAFs with hedgehog pathway inhibitor emerged as a potential approach to improve TNBC treatment outcome [196]. Recently, it was shown that the hedgehog signaling inhibitor sonidegib to downregulates the expression of CSC markers and increases the sensitivity of TNBC to paclitaxel in phase I clinical study (NCT02027376), thus improving patient survival and reducing metastasis in advanced TNBC [197]. Another inhibitor of hedgehog signaling, vismodegib (GDC-0449), was added to standard neoadjuvant chemotherapy in phase II clinical study (NCT02694224) due to its ability to inhibit breast CSC self-renewal and mammosphere formation [198]. The status of this clinical trial remains unknown.

#### 5.2.1. Reverting CAFs into Quiescent State

Therapies reverting CAFs to a quiescent state, such as targeting vitamin D receptor that represents a transcriptional suppressor of activated CAFs, have been shown to contribute to enhanced delivery of anticancer drug in animal models. In this sense, a phase I clinical trial (NCT00637897) investigated the effect of paricalcitol (19-nor-1,25-dihydroxyvitamin D2) in assisting chemotherapeutic drugs to more efficiently kill tumor cells. The study showed safety and feasibility in women with metastatic breast cancer receiving taxanes or ixabepilone [199].

All trans retinoic acid (ATRA) activates retinoic acid receptor (RAR)β and introduces CAFs into quiescent state by downregulating actin myosin contractility, thereby reducing CAF activities related to ECM remodeling and cell migration [200]. An Italian single-center randomized phase II clinical study (NCT04113863) is presently investigating preoperative activity of ATRA in HR+/HER2- early breast cancer in combination with non-steroidal aromatase inhibitor anastrozole.

#### 5.2.2. Targeting ECM

As desmoplasia and increased interstitial pressure exert compression on tumor vasculature impeding the effective distribution of chemotherapeutic agents into tumor tissue, the inhibition of ECM production by CAFs represents another CAF-targeted therapeutic strategy. For example, enzymatic activity of hyaluronidase can be used to modify tumor architecture for a more effective delivery of chemotherapeutics. In this sense, hyaluronidase PEGPH20 in combination with eribulin versus eribulin alone showed in phase II clinical trial (NCT02753595) improved antitumor effects in subjects with HER2-negative breast cancer [201]. Furthermore, angiotensin receptor blocker losartan, also known for inhibiting the production of collagen and hyaluronan and suppressing TGF-β-mediated fibrotic signaling, represents another agent targeting the ECM that showed some efficacy in the preclinical model of TNBC [202]. Losartan was included in the treatment protocol in a phase II clinical trial (NCT05097248) evaluating PD-1 inhibitor camrelizumab and liposomal doxorubicin in patients with advanced or locally advanced TNBC, who were previously treated with no more than one line of chemotherapy.

## 6. Conclusions

The accumulating evidence shows the importance of complex crosstalk between cancer cells, immune cells, fibroblasts, endothelial cells, molecular stromal components, and extracellular matrix components for tumor progression and response to therapy. The importance of TME for response to therapy is very pronounced in TNBC due to the lack of adequate molecular therapeutic targets confined to tumor cells and the absence of effective treatment options except for chemotherapy. Although multiple clinical trials have recently implied the benefits of introducing immunotherapy into the treatment of TNBC, the response rates remained low due to activation of additional suppressive mechanisms in TME during the course of therapy that undermine the therapeutic benefit. In this sense therapeutic targeting of the TME and its factors, including cytokines, enzymes, metabolites, chemokines, and signaling pathways in TME, represents potential complementary therapeutic strategy in TNBC. Furthermore, CAFs emerge as relevant targets due to their multiple roles in tumor initiation and progression. Moreover, CAFs confer cancer cell resistance to therapy, as they orchestrate immunosuppressive TME by interacting with immune cells and play a crucial role in the formation of ECM that serves as a physical barrier for therapeutic drugs and immune cells from reaching the tumor.

However, based on the recent data obtained from clinical investigations, the most of currently available agents targeting cytokines, cytokine receptors, prostaglandin receptors, IDO-1 activity and CAF-related targeting agents have reached phase I/II clinical trials. The leading number of developed pharmacological agents and clinical trials investigating them was related to blockade of TGF-β signaling and IDO inhibition. For efficient clinical application, further development of therapeutics with high specificity for their target in TME is needed, as well as experimental evaluations of strategies that simultaneously target several components in the TME. Furthermore, better understanding of the interactions between cells and molecular components in the TME is crucial for discovering novel druggable targets related to molecular and immunological aspects of TNBC.

## Figures and Tables

**Table 1:** Summary of clinical trials targeting tumor microenvironment in TNBC.

Therapeutic Target	Agent	Therapy Design	Group of Patients	Clinical Trial Number and Phase	Status
**TGF-β pathway**					
TGF-β RI (ALK5)	PF-06952229	Monotherapy	Advanced solid tumors	NCT03685591 Phase I	Terminated
TGF-β RI (ALK4/ALK5)	Vactosertib (TEW-197)	Monotherapy	Advanced solid tumors	NCT02160106 Phase I	Completed
TGF-β RI (ALK1)	PF-03446962	Monotherapy PF-03446962 +bavacizumab	Advanced solid tumors	NCT00557856 Phase I	Completed
TGF-β RI (ALK5)	LY3200882	Monotherapy LY3200882+ nab-Paclitaxel LY3200882+anti-PD-L1 (LY3300054)	Patients with solid tumors	NCT02937272 Phase I	Active, not recruiting
TGF-β RI (ALK5)	galunisertib monohydrate (LY2157299)	Monotherapy	Advanced refractory solid tumors	NCT02423343 Phase Ib	Completed
TGF-β RII and PD-L-1	bifunctional antibody SHR-1701	Monotherapy	Metastatic or locally advanced solid tumors	NCT03710265 Phase II/III	Unknown status
TGF-β RII and PD-L1	bifunctional antibody bintrafusp alfa	Monotherapy	Patients with Metastatic TNBC	NCT03579472 Phase Ib/II	Terminated
TGF-β RII and IL-15Rα	bifunctional antibody HCW9218	Monotherapy	Advanced solid tumors	NCT05322408 Phase I	Active, not recruiting
**IL-6/JAK/STAT-3 pathway**					
IL-6 R	tocilizumab	Monotherapy	Steroid-dependent immune-related adverse events (irAEs)	NCT04375228 Phase II	Active, not recruiting
STAT3	OPB-51602	Monotherapy	Advanced Cancer	NCT01423903 Phase I	Completed
STAT3	VVD-130850	Monotherapy	Advanced solid and hematologic tumors	NCT06188208 Phase I	Recruiting
STAT3	IMX-110	IMX-110 + tislelizumab (anti-PD-1)	Advanced solid tumors	NCT05840835 Phase I/IIa	Recruiting
STAT3	AZD9150	AZD9150 + durvalumab	Advanced solid tumors	NCT02499328 Phase Ib/II	Active, not recruiting
		AZD9150 + durvalumab	Advanced solid tumors	NCT03394144 Phase I	Completed
**IL-8**					
IL-8	BMS-986253 (HuMax-IL-8)	Monotherapy	Advanced solid tumors	NCT02536469 Phase Ib	Completed
**CXCL12/CXCR4 pathway**					
CXCR4	Bilixafortide (POL6326)	Bilixafortide + eribulin	Relapsed TNBC and hormone refractory ER-positive metastatic BC	NCT01837095 Phase I	Completed

Activin receptor-like kinase (ALK), triple-negative breast cancer (TNBC), programmed death ligand-1(PD-L1), estrogen receptor (ER), breast cancer (BC).

**Table 2:** Summary of clinical trials targeting IDO1 in TNBC.

Agent	Therapy Design	Group of Patients	Clinical Trial Number and Phase	Status
BMS-986205 (Linrodostat)	BMS-986205+ nivolumab BMS-986205 + nivolumab + ipilimumab BMS-986205 + ipilimumab	Advanced malignant tumors	NCT02658890 Phase I/IIa	Completed
BMS-986205	BMS-986205+ nivolumab/relatlimab/ipilimumab	Advanced malignant tumors	NCT03335540 (ADVISE) Phase I	Completed
BMS-986205	BMS-986205 + relatlimab (anti-LAG-3) + nivolumab	Advanced malignant tumors	NCT03459222 Phase I/II	Completed
Epacadostat	Epacadostat + pembrolizumab + chemotherapy	Advanced or metastatic solid tumors	NCT03085914 (ECHO-207/KEYNOTE-723) Phase I/II	Completed
LY3381916	LY3381916 + antiPD-L1 (LY3300054)	Solid tumors	NCT03343613 Phase I	Terminated
E7046	Monotherapy	Selected advanced malignancies	NCT02540291 Phase I	Terminated
HTL0039732	Monotherapy HTL0039732 + atezolizumab	Advanced solid tumors	NCT05944237 Phase Ib/IIa	Recruiting
Buparlisib (AN0025)	Buparlisib (AN2025) + AN0025 AN2025 + AN0025 + atezolizumab	Advanced solid tumors	NCT04975958 Phase Ia	Completed
eganelisib (IPI-549)	Monotherapy PI-549 + nivolumab	Advanced solid tumors	NCT02637531 Phase I/Ib	Unknown status
IPI-549	IPI-549+ tecentriq (atezolizumab) + nab-paclitaxel	Front-line TNBC	NCT03961698 MARIO-3 Phase II	Active, not recruiting

Triple-negative breast cancer (TNBC).

**Table 3:** Summary of clinical trials targeting PGE2 in TNBC.

Agent	Therapy Design	Group of Patients	Clinical Trial Number and Phase	Status
E7046	Monotherapy	Advanced malignancies	NCT02540291 Phase I	Terminated
HTL0039732	Monotherapy HTL003973+ atezolizumab	Advanced solid tumors	NCT05944237 Phase I/IIa	Recruiting
AN0025	AN0025 + AN2025 (pan-class I PI3K inhibitor) AN0025 +atezolizumab	Advanced malignant tumors	NCT04975958 Phase Ia	Completed

Triple-negative breast cancer (TNBC).

**Table 4:** Summary of clinical trials targeting PI3K-γ in TNBC.

Agent	Therapy Design	Group of Patients	Clinical Trial Number and Phase	Status
Eganelisib (IPI-549)	Monotherapy	Advanced solid tumors	NCT02637531 Phase I/Ib	Terminated
IPI-549	IPI-549 + nabpaclitaxel + atzolizumab	Front-line triple TNBC	NCT03961698 Phase II	Unknown status

Triple-negative breast cancer (TNBC).

**Table 5:** Summary of clinical trials targeting cancer-associated fibroblasts in TNBC.

Activity/Mechanism	Agent	Therapy Design	Group of Patients	Clinical Trial	Status
**Targeting FAP** **α**					
FAPα- targeted CD40 agonist antibody	RO7300490	Monotherapy RO7300490 + atezolizumab	Advanced and/or metastatic solid tumors	NCT04857138 Phase I	Completed
**Targeting FGFR signaling**					
Pan FGFR 1-4 inhibitor	Erdafitinib	Monotherapy	Advanced solid tumors	NCT04083976 RAGNAR Phase II	Completed
		Erdafitinib + Fulvestrant+ Palbociclib	ER+/HER2-/FGFR-amplified BC	NCT03238196 Phase I	Active, not recruiting
**Targeting Hedgehog signaling**					
Smoothened receptor (SMO) antagonist	Sonidegib (LDE225)	Sonidegib + docitaxel	Advanced TNBC	NCT02027376 EDALINE Phase Ib	Completed
Inhibitor of SMO	Vismodegib (GDC-0449)	Vismodegib + standard neoadjuvant chemotherapy	TNBC	NCT02694224 Phase II	Unknown status
**Reverting CAFs into quiescent state**					
Vitamin D receptor agonist	19-nor-1,25-dihydroxyvitamin D2	19-nor-1,25-dihydroxyvitamin D2 + standard Neoadjuvant Chemotherapy	Metastatic Breast Cancer	NCT00637897 Phase I	Completed
RARβ agonist	All trans retinoic acid (ATRA)	ATRA + non-steroidal aromatase inhibitor anastrozole	Hormonal receptor+/HER2- early BC	NCT04113863 Phase II	Recruiting
**Targeting ECM**					
Inhibition of hyaluronidase activity	PEGPH20 PEGylated Recombinant Human hylaronidaze	PEGPH20 + eribulin mesylate	HER2-, high-hyaluronan metastatic BC	NCT02753595 Phase Ib/II	Terminated
Inhibition of colagen and hyaluron production	Losartan (angiotensin receptor blocker)	Losartan + camrelizumab +doxorubicin	TNBC treated with no more than 1 Prior line of chemotherapy	NCT05097248 Phase II	Unknown status

(TNBC), estrogen receptor (ER), estrogen receptor (ER), breast cancer (BC), human epidermal growth factor receptor2 (HER2), fibroblast growth factor receptor (FGFR), retinoic acid receptor (RAR).

## Data Availability

No new data were created or analyzed in this study. Data sharing is not applicable to this article.
